# Price elasticity and affordability of aerated or sugar-sweetened beverages in India: implications for taxation

**DOI:** 10.1186/s12889-022-13736-2

**Published:** 2022-07-17

**Authors:** Rijo M. John, Fikru T. Tullu, Rachita Gupta

**Affiliations:** 1 Rajagiri College of Social Sciences, Kochi, Kerala 683104 India; 2 Non Communicable Diseases, World Health Organization, New Delhi, India; 3 National Professional Officer (Nutrition), World Health Organization, New Delhi, India

**Keywords:** Sugar-sweetened beverages, India, Price elasticity, Taxation, Affordability

## Abstract

**Background:**

The sale of aerated or sugar-sweetened beverages (ASBs) has been consistently growing in India which has also experienced a major increase in non-communicable diseases. This study estimates the price elasticities of ASBs by different household-income groups in India and examine the trends in their affordability.

**Methods:**

The price elasticity for ASBs were estimated using a nationally representative household sample survey on consumption of ASBs in India and with Deaton’s method which is robust to self-reported household expenditure surveys. Trends in affordability of ASBs were estimated using relative income price (RIP) which measured the proportion of per capita gross domestic product (GDP) required to purchase 100 L of ASBs in a given year. The elasticity parameters were used to estimate the incremental tax needed for a 10% reduction in ASB consumption.

**Results:**

The own-price elasticity of ASBs is − 0.94 in the overall sample and varied between − 1.04 to − 0.83 from low- to high-income households. There has been an annual average decline of about 6.8% in RIP of ASBs or an increase in their affordability over the last 13 years. Increasing the compensation cess on ASBs under the current Goods and Services Tax (GST) to 29%, will have the effect of decreasing ASB consumption by 10% and increasing the tax revenue by about 27%.

**Conclusion:**

The taxation policy on ASBs in India has largely been ineffective at increasing the real retail prices of ASBs as a result of which ASB consumption grew. ASBs should be classified along with other unhealthy products like tobacco and alcohol as *demerit products* for the purpose of taxation and their taxes should be regularly increased sufficiently enough to compensates for both general price inflation and income growth so as to decreases their affordability.

## Background

The consumption of sugar, along with tobacco and alcohol are major risk factors for Non-communicable Diseases (NCDs) and it disproportionately affects people with low socioeconomic status in low-income countries [1]. Unhealthy diet, in particular, aerated or sugar-sweetened beverages (ASBs), can result in several health problems [2–6] including obesity, type 2 diabetes, high blood pressure, asthma, and it can result in premature deaths and disabilities while negatively impacting productivity and economic growth. Among the Indian women and men, it is known that dietary risks contributed to 51.8% and 59.4% of the total disability-adjusted life years from cardiovascular diseases, respectively [7]. The prevalence of childhood obesity and overweight in India range between 4% to 12% and 6% to 25%, respectively, while more than 21% of adults are overweight [8]. NCDs accounted for 61.8% of all deaths in India in 2016, up from only 37.9% in 1990 [9]. A substantial body of literature clearly shows that reducing consumption of ASBs and/or substituting to non-caloric beverages reduces obesity [10, 11].

Notwithstanding their established health consequences, the sales of aerated drinks have seen a 22.5% increase and that of all soft drinks increased by 24.8% from 2016 to 2019 in India [12]. Yet, the annual purchases of ASBs for consumption at home are estimated to be quite low at 1.1 L per capita in 2017 [13]. Per capita annual consumption of ASBs alone was 1 L and 0.46 L in urban and rural India, respectively [14].

Research has also shown that higher prices for sugary foods would significantly reduce consumption of such products and prevent the rise in overweight and obesity among adults and children [15–19]. Studies also show ASB taxation has the effect of reducing consumption particularly when baseline consumption levels are high [12, 20–22]. However, the effectiveness of ASB taxation is unclear in countries with low baseline consumption [12]. Given that ASB consumption is relatively low in India, it is important to estimate if taxation has a significant effect on ASB consumption. The only study [23] estimating the price elasticity of ASBs in India, used household expenditure data and found that the own-price elasticity of ASBs was about − 0.94. However, this study used self-reported household expenditures and quantities to estimate price elasticities and did not adjust for the measurement errors and quality variations in unit values which are used as proxies for prices while estimating price elasticities. As a result, the estimated price elasticity may be biased [24].

This study aims to examine the own-price elasticity of ASBs in India and its cross-price elasticity with select beverages such as juice, milk & tea. The ASBs, in this study include bottled/canned aerated drinks with or without added sugar and sugar-sweetened beverages which may or may not be aerated. Fruit juices with or without added sugar which are not bottled/canned, however, are not part of this although the elasticities for them are captured in the study as a separate category called *juice*. The study offers a significant methodological improvement over the only existing study on price elasticity of ASBs in India [23] by using a more robust econometric method of price elasticity estimation with household expenditure surveys which is explained in detail under the methods section. In addition, it also examines the trends in affordability of the top aerated drink brands (Coca-Cola, Pepsi, and Thumps Up) in India for the first time.

## Methods

### Estimating price elasticity of ASBs

The own- and cross-price elasticity for ASBs and other beverages such as juice, milk, and tea were estimated using the last two quinquennial rounds—66th (2009–10) and 68th (2011–12)—of household consumption data by the National Statistical Office (NSO) [14, 25]. Although there were other surveys after this, they were not for examining the household consumption expenditures. The 68th round survey was done without the usual interval of 5 years in between different quinquennial surveys because the period of the 66th round survey was considered to be a “non-normal” year. Hence, for this study, we decided to use both the rounds and treat them as a pooled cross-section. The unit values in both these surveys were adjusted for inflation across the two rounds and the eight sub-rounds over which the surveys spread using the consumer price index for food inflation with the base year 2012. The 66th and 68th rounds collected consumption information from a nationally representative sample of 100,794 and 101,651 households spanning over 12,691 and 12,737 villages/urban blocks, respectively, spread across the length and breadth of India. The quantity consumed and expenditure at the household level were canvased for beverage items such as ASBs, milk, tea, coffee, mineral water, and fruit juices. As the prevalence of coffee and mineral water consumption is quite low (1%) in these surveys, these were excluded from our analysis.

Since the survey provides both quantity and value of consumption, one can compute unit values (expenditures divided by quantity) that can be used as proxies for prices [26]. However, the price and choice of quality affect unit values [24, 27]. When prices rise, consumers shade down both quality and quantity and, as a result, unit values tend to vary less than the prices. Hence, using unit value as a proxy for the price can potentially overstate the effect of price on quantities (quality shading). In addition, the measurement errors in either the quantity or expenditure will get carried over to the unit values. It is important to correct both these while using unit values as a proxy for prices. Deaton proposes a method of doing this to estimate a system of demand equations similar to the Almost Ideal Demand System [28]. This method has been used to estimate the price elasticities of demand for various products including tobacco products in several countries [29] and ASBs in Guatemala [30]. The only existing study estimating the own-price elasticity for ASBs in India [23], however, uses unit values as proxies for prices, but without correcting for both quality shading and measurement errors.

Deaton uses a two-equation system to estimate the own- and cross-price elasticities:

1 $${lnv}_{hc}={\alpha}^1+{\beta}^1{lnx}_{ic}+{\boldsymbol{\gamma}}^{\mathbf{1}}{\boldsymbol{Z}}_{\boldsymbol{h}c}+\psi ln{\pi}_c+{u}_{hc}^1$$

2 $${w}_{hc}={\alpha}^0+{\beta}^0\mathit{\ln}{x}_{ic}+{\boldsymbol{\gamma}}^{\mathbf{0}}{\boldsymbol{Z}}_{\boldsymbol{h}c}+\theta {ln\pi}_c+\left({f}_c+{u}_{hc}^0\right)$$

*lnv*_*hc*_ is the log of the unit value for household *h* in cluster *c*, where the cluster is typically a village in rural areas or an urban block in urban areas in the survey. *w*_*hc*_ represents the share of expenditure on ASBs or other beverages (juice, milk, and tea) in total household expenditure for household *h* in cluster *c*. *lnx*_*hc*_ is the log of total household expenditure over the relevant reference period. ***Z***_***hc***_ is a vector of household-specific characteristics, which include variables such as logarithm of household size, number of children below 18 years, household type indicating the employment status, social group indicating the caste of households, religion of household, gender of household head, and average years of education of household. *f*_*c*_ is a cluster fixed effect and treated as an error in addition to the error term ${u}_{hc}^0$ in eq. 2, while ${u}_{hc}^1$ is the standard regression error term. Both ${u}_{hc}^0$ and ${u}_{hc}^1$, however, incorporate any measurement errors in budget shares and unit values, apart from the usual unobservables. The detailed description of Deaton’s methods and the steps involved in its estimation are available elsewhere [26, 27, 29]. The elasticity estimation was done using a modified version of the Stata codes from Deaton [27] and we used the statistical software Stata (ver. 15.0) [31].

Deaton’s model assumes that there is no price variation within each cluster and, instead, the model exploits the genuine price variation that exists between clusters. Any variation in unit values observed within the cluster is due to differences in quality consumed as well as measurement errors both of which are corrected for in the model. The assumption holds well for household survey data that is used here since the clusters in this survey represent households residing with good geographical proximity as they are typically a village in rural areas or an urban block in urban areas. Moreover, the survey is done around the same time for all households in a given cluster. It is also quite reasonable to assume that the prices (unit values) across clusters genuinely vary due to the differences in cost of transportation as well as differences in state-level sales taxes on ASBs across states in India. An analysis of the variance of unit values across clusters indicated that the genuine price variation between clusters explained 69% of the variation in unit values as indicated by the *R*^*2*^ values from the regression of log unit values on cluster dummies.

After dropping households that do not consume any ASBs and keeping only clusters having at least 2 households consuming ASBs, we were left with a total of 2322 clusters giving a good enough sample size to implement Deaton’s model. Households were divided into three income tertiles—low, middle, and high—using total household expenditures as a proxy for income, and elasticities were estimated separately for each group. Deaton’s model was not originally designed to estimate the elasticities by income groups. The assumption in Deaton’s model is that all households within the same cluster face the same prices. The model codes were adapted such that households in each income group face the same price as long as they are drawn from the same cluster. This would take care of a potential endogeneity in average prices that might arise if households from different income groups face different average prices despite being in the same cluster. The estimated income-group-wise price elasticities were used to simulate the required tax increases to achieve a 10% reduction in consumption under alternative tax-pass through assumption.

The paper also undertakes an analysis of the current taxation of ASBs in India with the intention of providing some fiscal policy recommendations. Before the introduction of the Goods and Services Taxation (GST) in July 2017, the indirect taxes on ASBs consisted of a uniform excise duty applied by the central government and a value-added tax (VAT) applied by various state governments which varied from one state to the other. First, we did a trend analysis of pre-GST taxation of ASBs which examined the trends in both excise tax revenue and changes in excise tax rates on ASBs. The excise duty rates were collected from different volumes of the *central excise tariffs of India* [32] and the excise revenue from the Central Board of Indirect Taxes and Customs (CBIC), Government of India. The GST legislation in July 2017 subsumed both excise and VAT into a uniform tax rate across the country. For the second part of the tax analysis, we set up a simple simulation in Microsoft Excel. The simulation used baseline sales volume of all aerated drinks [33], tax revenues from aerated drinks, and the estimated own-price elasticity coefficients of ASBs from this paper. It estimated the incremental tax needed for an arbitrary 10% reduction in ASB consumption assuming different tax pass-through scenarios under the new GST regime. Although the evidence suggests that producers tend to fully pass through tax increases, sometimes passing more than 100% of tax increases to the retail prices [21, 34–37], we used tax passthrough scenarios of 50%, 75%, and 100% for the simulation. The impact of taxes on consumption and tax revenue was estimated separately for households of different income groups. For this we used own-price elasticities for each income group as shown in Table 3 and the share of each income group in consumption of ASBs estimated from the household consumption survey [38]. The simulation would estimate the necessary increase in retail price of ASB brought about by taxation, assuming a given tax passthrough to price scenario, to affect an overall 10% reduction in its consumption. The actual percentage reduction in consumption experienced by each income group, however, would be different depending on the own-price elasticity of each.

### Estimating affordability of ASBs

The income of the consumer and the price of the product are two main variables that determine affordability. When the real price of a commodity decreases, a consumer can purchase more quantities of that commodity with the same amount. Income growth enables the purchase of more quantities of the same goods by spending the same share of income as before. Relative income price (RIP) is a measure that is widely used in the literature [39, 40] to measure the true affordability of products over time that capture both these dimensions. RIP, in this context, is defined as the percentage of per capita gross domestic product (GDP) required to purchase 100 L of ASBs in a year. Some studies [41] on affordability also uses wages or average household expenditures in the denominator although per capita GDP is the preferred and most widely used variable. A higher RIP in a year compared to a previous year means, ASBs have become less affordable and vice versa.

Retail prices of popular aerated drinks are available from the labor bureau [42] which collects these monthly from about 80 centers spread across most states in India. These retail price data and the per capita GDP data from the Reserve Bank of India (RBI) [43] were used to estimate RIP for 13 years from 2006/07 to 2018/19 for the three most popular aerated drinks in India, namely, *Coca-Cola*, *Pepsi,* and *Thums up*. The percentage change in RIP (or change in affordability) every year was also decomposed into an effect due to a change in real prices and an effect due to a change in income. For this purpose, the percentage change in real price was subtracted from the percentage change in RIP to decompose and derive the effect of an income change and price change on affordability separately [39, 40].

## Results

Table 1 presents summary data on the consumption of beverages by Indian households. The purchase of ASBs is very low and it has a strong income gradient. While at least 10% of high-income households consume ASBs, only about 1% of low-income households do so. The per capita quantity of consumption is also directly proportional to the household income. Consumption of fruit juices is also limited with only 3% prevalence. As a share of the household budget, low-income households spent a relatively larger share on the purchase of ASBs compared to their high-income counterparts.

**Table 1:** Household consumption of different beverages in India

	66th Round (2009–10)				68th Round (2011–12)			
	Full Sample	Low Income	Middle Income	High Income	Full Sample	Low Income	Middle Income	High Income
**The proportion of households purchasing**								
ASB: bottled/canned	4.0%*	0.9%*	3.2%*	10.7%*	3.9%*	1.0%*	3.1%*	10.0%*
Fruit juice and shake	3.1%*	0.8%*	2.0%*	8.5%*	2.7%*	0.8%*	1.9%*	6.8%*
Milk: liquid	78.9%*	64.4%*	86.9%*	93.7%*	80.2%*	65.4%*	88.4%*	94.5%*
Tea: cups	41.7%*	36.5%*	44.0%*	48.0%*	41.1%*	33.5%*	43.5%*	50.9%*
**Average quantity purchased by household per month**								
ASB: bottled/canned (litre)	4.41*	2.0*	2.8*	5.4*	5.7*	2.3	7.9	5.4*
Fruit juice and shake (litre)	3.83*	2.7	2.8*	4.3*	3.6*	2.0*	2.8*	4.2*
Milk: liquid (litre)	25.39*	12.8*	24.1*	42.1*	25.6*	13.6*	24.0*	41.5*
Tea: cups (no.)	48.27*	40.3*	47.5*	59.8*	43.1*	35.38*	41.8*	53.3*
**Average expenditure incurred by household per month (base = 2012 June)**								
ASB: bottled/canned (INR)	187.38*	85.8*	124.0*	227.7*	176.3*	91.5*	133.8*	208.7*
Fruit juice and shake (INR)	220.93*	107.1**	131.2*	266.0*	214.8*	98.4*	148.0*	264.2*
Milk: liquid (INR)	620.67*	288.7*	562.8*	1091.9*	692.4*	333.3*	626.8*	1194.6*
Tea: cups (INR)	172.61*	125.3*	164.9*	245.1*	178.7*	126.5*	169.3*	247.6*
**Average unit value (INR/unit) (base = 2012 June)**								
ASB: bottled/canned (INR/litre)	49.36*	47.7	51.5	48.8	45.3*	45.9	46.0	44.9*
Fruit juice and shake (INR/litre)	65.31*	49.6**	57.9*	70.0*	69.4*	61.0	63.4*	73.5*
Milk: liquid (INR/litre)	24.47*	23.1*	24.1*	26.6*	27.0*	25.2*	26.9*	29.2*
Tea (INR/cup)	3.74*	3.3*	3.7*	4.4*	4.4*	3.9*	4.4*	5.0*
**Average budget share devoted by household**								
ASB: bottled/canned	1.9%*	2.7%*	2.2%*	1.7%*	1.5%*	2.3%*	1.8%*	1.3%*
Fruit juice and shake	2.1%*	3.5%*	2.3%*	1.8%*	1.7%*	2.5%**	2.0%*	1.4%*
Milk: liquid	9.9%*	9.6%*	10.3%*	9.8%	8.5%*	8.4%*	8.7%*	8.2%**
Tea: cups	3.4%*	4.7%*	3.0%*	2.2%*	2.6%*	3.5%*	2.3%*	1.7%*

Single star (*) and double stars (**) indicate levels of significance at 1 and 5%, respectively; *INR* - Indian rupee

Source: Estimated from National Sample Survey data, Government of India, 2012, 2014

### Price elasticity of ASBs

Table 2 presents own- and cross-price elasticities for different ASBs and select beverages. The overall own-price elasticity was − 0.94 for ASBs. It means, for every 10% increase in price for ASBs, a decrease in consumption by 9.4% is expected among ASB-consuming households, with everything else remaining the same. Juice, on the other hand, was relatively more inelastic with elasticity at − 0.55 although it was not statistically significant. The cross-price elasticity coefficients were positive with respect to juice and tea implying substitution of ASBs with these products while it was negative with respect to milk implying a complementarity. Separate rural-urban regressions show the own-price elasticity coefficient was marginally bigger in rural India compared to urban India implying that rural India is relatively more price responsive towards ASBs.

**Table 2:** Own- and cross-price elasticity estimates

	ASB	Juice	Milk	Tea
**Full sample**				
ASB	−0.944 (0.073)*	0.206 (0.055)*	−1.807 (0.188)*	0.271 (0.054)*
Juice	1.533 (0.412)*	**−0.550 (0.385)**	− 0.130 (1.907)	− 0.164 (0.315)
Milk	− 0.339 (0.035)*	− 0.001 (0.047)	− 0.389 (0.064)*	0.208 (0.026)*
Tea	0.541 (0.107)*	−0.041 (0.084)	2.250 (0.284)*	−0.989 (0.116)*
**Rural Sample**				
ASB	−1.000 (0.128)*	0.166 (0.092)***	−2.008 (0.354)*	0.324 (0.099)*
Juice	2.013 (1.132)***	**−0.486 (0.762)**	0.904 (6.254)	−0.293 (0.838)
Milk	−0.391 (0.068)*	0.016 (0.098)	−0.312 (0.123)**	0.255 (0.049)*
Tea	0.701 (0.215)*	−0.050 (0.148)	2.902 (0.546)*	−1.273 (0.217)*
**Urban Sample**				
ASB	−0.992 (0.094)*	0.200 (0.066)*	−1.539 (0.231)*	0.239 (0.061)*
Juice	1.161 (0.387)*	**−0.581 (0.441)**	−0.556 (1.708)	0.039 (0.306)
Milk	−0.351 (0.052)*	− 0.019 (0.066)	− 0.196 (0.085)**	0.191 (0.036)*
Tea	0.457 (0.116)*	0.016 (0.100)	1.631 (0.303)*	−0.858 (0.106)*

The elasticity in row *i*, column *j* estimates the effect of a change in the price of good *j* on the quantity demanded of good *i.* Values in parentheses are the bootstrapped standard errors calculated by making 1000 draws from the second stage cluster-level regressions. Assuming the estimates follow a normal distribution, the coefficients with *, **, and *** imply levels of significance at 1, 5, and 10%, respectively

Table 3 presents the estimates of own- and cross-price elasticities for ASBs and other beverages across different income groups. One can see a clear income gradient for the consumption of ASBs in India with the coefficient of own-price elasticity at − 1.04 for the low-income households and − 0.83 for the high-income households. The coefficient, although larger, was not statistically significant for the middle-income households. Many of the cross-price elasticity coefficients are not statistically significant.

**Table 3:** Own- and cross-price elasticity estimates: income groups

	ASB	Juice	Milk	Tea
**Low-income Households**				
ASB	−1.035 (0.075)*	0.073 (0.090)	−0.439 (0.321)	− 0.032 (0.081)
Juice	0.529 (0.688)	**−0.064 (1.745)**	−0.964 (5.407)	0.816 (0.944)
Milk	−0.096 (0.061)	−0.022 (0.134)	**− 0.124 (0.125)**	0.118 (0.055)**
Tea	−0.059 (0.144)	0.196 (0.222)	1.189 (0.517)**	**−0.045 (0.256)**
**Middle-income Households**				
ASB	−0.913 (0.072)*	0.214 (0.083)**	−1.268 (0.241)	0.105 (0.042)**
Juice	1.616 (0.641)**	**1.241 (1.208)**	−3.294 (4.402)	−0.548 (0.501)
Milk	−0.273 (0.051)*	−0.087 (0.122)	0.345 (0.196)***	0.093 (0.039)**
Tea	0.289 (0.114)**	−0.187 (0.175)	1.223 (0.488)**	−0.723 (0.108)*
**High-income Households**				
ASB	−0.832 (0.079)*	0.012 (0.072)	−2.033 (0.236)*	0.249 (0.056)*
Juice	0.083 (0.566)	**−0.408 (0.472)**	2.763 (1.922)	−0.212 (0.335)
Milk	−0.452 (0.053)*	0.081 (0.055)	**−0.150 (0.142)**	0.156 (0.046)*
Tea	0.433 (0.097)*	−0.046 (0.075)	1.198 (0.356)*	−0.869 (0.112)*

The elasticity in row *i*, column *j* estimates the effect of a change in the price of good *j* on the quantity demanded of good *i.* Values in parentheses are the bootstrapped standard errors calculated by making 1000 draws from the cluster-level regressions. Assuming the estimates follow a normal distribution, the coefficients with *, **, and *** imply levels of significance at 1, 5, and 10%, respectively

### Affordability of ASBs

**Fig. 1:**
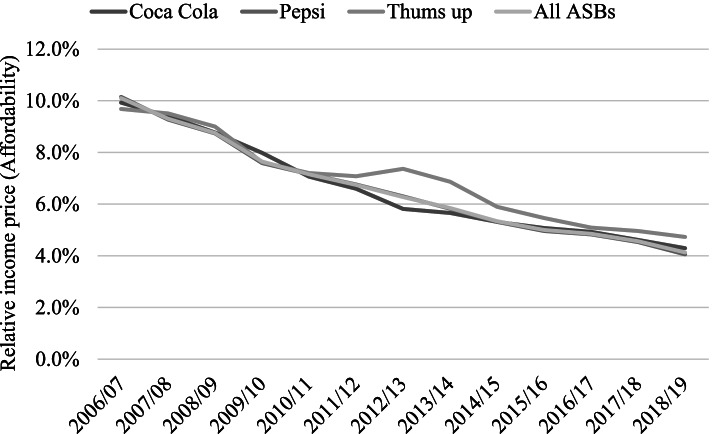
Trends in affordability of select aerated or sugar-sweetened beverages (ASBs) in India. * Relative income price shows the % of per capita income required to purchase 100 L of ASBs in a year. Source: Retail price data taken from “Retail Prices from Consumer Price Index for Industrial Workers” Labour Bureau (2020) and per capita GDP data from the Reserve Bank of India (RBI) (Reserve Bank of India, 2017)

Figure 1 shows the trends in affordability of three popular aerated drinks—*Coca-Cola*, *Pepsi,* and *Thums up*—in India for which retail prices were available. There were 10,333 price observations over the 13 years for all the aerated drinks combined in the retail price database. Out of this, *Pepsi* constituted 78.5% (8107) of the sample followed by *Coca-Cola* and *Thums up* with 15.4% (1590) and 4.6% (477) observations, respectively together contributing 98.5% of the sample. The share of these three brands in the total on-trade volume of carbonates sold in India was, however, only 40% in 2017 [33]. The affordability has consistently increased during the years 2006/07 to 2018/19 as shown by a consistently falling RIP graph. For example, while it took 10.1% of the annual per capita income to purchase 100 L of ASBs in the year 2006/07, it took only 4.1% to purchase the same amount of ASBs in 2018/19. There has been an annual average decline of about 6.8% in RIP of ASBs in the past 13 years suggesting a steady increase in affordability. Except for the year from 2015 to 16 to 2016–17 where the decline in RIP was about 3%, in all other years, the decline was about 6% or higher.

The per capita GDP has shown a geometric mean growth of 4.9% over the period 2006–07 to 2018–19 and that has played a major role in increasing the affordability of ASBs. However, whether it is this consistent growth in per capita GDP or the drop in real retail prices that contributed more heavily to the increase in affordability needs further examination through decomposition of their relative contribution.

**Fig. 2:**
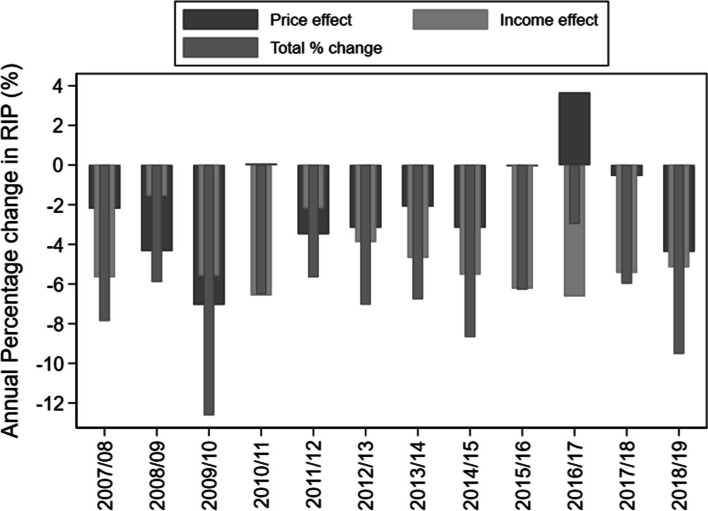
Decomposition of the change in relative income price (RIP)*. due to price and income. * Relative income price shows the % of per capita income required to purchase 100 L of SSBs in a year

Figure 2 decomposes the annual percentage change in RIP into an effect due to changes in real prices and an effect due to changes in real per capita income. The thinnest bars indicate the total annual percentage change in RIP. If the bar is negative, it means the RIP has decreased compared to the previous year or the ASB has become more affordable and vice versa. The affordability increased in all the years due to a combination of increases in per capita income and a decrease in real prices except 2016–17. In the year 2016–17, a 3.7% increase in the real price compensated the negative effect of a 6.6% income increase on RIP to a great extent and, as a result, the net decline of RIP has only been 3%. In all the other years, both the price decrease as well as income increase contributed to rising affordability.

### Taxation of ASBs in India

**Fig. 3:**
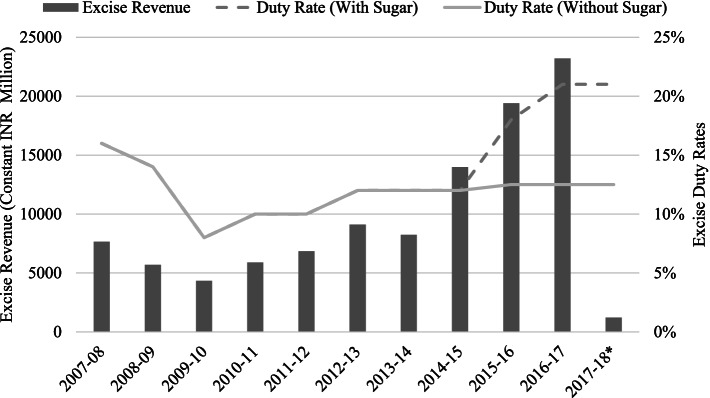
Trends in excise tax revenue and duty rate on aerated or sugar-sweetened beverages in India. * The revenue for the financial year 2017–18 reflects only the revenue collected for the first quarter as the goods and services tax (GST) was introduced from the second quarter onwards. Source: Directorate of Data Management, Central Board of Indirect taxes and Customs, Government of India

Figure 3 shows the trends in excise duty rates as well as tax revenue (in constant INR, base 2017/18 = 100) on ASBs. The duty rates remained the same for all ASBs at 12% until 2014–15 after which the rate of ASBs with added sugar started diverging from those without added sugar and has since gone up. The excise duty rates for ASBs with added sugar increased to 18% in 2015/16 and to 21% in 2016/17 while that for ASBs without added sugar remained at 12.5%. Interestingly, the year 2016/17 also saw a 3.7% increase in the real price significantly compensating the negative effect of income increase on affordability as seen earlier. In all the other years, the relatively low excise duty rate changes have not contributed to an increase in the real price of ASBs. There is a positive correlation between the excise revenue collection and the excise duty rates with a positive correlation coefficient of 0.42.

The VAT varied across states and averaged 15.75% for the year 2017–18 at the time GST was introduced. The GST council fixed a statutory (exclusive) *ad-valorem* GST rate of 28% and an additional duty of 12% known as *compensation cess* on aerated drinks. The total statutory rate of 40% effectively meant the total tax burden of ASBs—tax as a percentage of the tax-inclusive retail price—is about 28.6%. This has not been changed for more than 4 years since the introduction of GST and might have contributed further to the increasing affordability.

Table 4 shows the incremental taxes required for achieving a 10% reduction in ASB consumption under different tax pass-through scenarios. Since changing the GST rate itself is less feasible compared to changing the additional compensation cess, the simulation translates the required tax increase to an increase in compensation cess. Assuming a 100% pass-through of taxes, it takes about a 41% increase in the present tax to impact a 10% decline in consumption. The 41% increase in absolute tax amount translates to a 29% compensation cess. It is only an additional 17% cess over the current 12%. If the pass-through is only 75%, it would take a compensation cess of 38% (an additional 26%) to make a similar impact on consumption. A 29% compensation cess will have the effect of increasing the GST revenue from aerated drinks by 27% (or INR 25.8 billion) and it will only lead to a nominal price increase of SSBs from the current INR 60 to INR 67.1 per liter.

**Table 4:** Required incremental tax for a 10% consumption reduction in ASBs

	Baseline	Tax pass-through scenario		
		50%	75%	100%
Average retail price (INR/liter)	60	67.1	67.1	67.1
Tax component in retail price (INR/liter)	17.2	31.3	26.6	24.2
Consumption volume (million liter)	5568	5011	5011	5011
Estimated GST revenue (INR million)	95,547	156,707	133,135	121,350
Tax burden (Tax as % of the retail price)	28.6%	47%	40%	36%
Changes (in percentage)				
Consumption	–	−10%	−10%	−10%
Tax Revenue	–	64%	39%	27%
Prices	–	12%	12%	12%
The required increase in tax (%)	–	82%	55%	41%
Absolute tax increase (INR/liter)	–	14	9	7
Required compensation cess rate	–	59%	38%	29%

## Discussion

This study estimates price elasticity of ASBs, analyze the trends in their affordability, and use the elasticity coefficients to estimate the incremental taxes needed under the current GST for a 10% reduction in ASB consumption. The study found that for every 10% increase in price for ASBs, its consumption decreases by 9.4%, with everything else remaining the same. Juice and tea were found to be substitutes for ASBs with positive cross-price elasticities. Whereas, the negative cross-price elasticity with milk implied complementarity. While it is intuitive to think that tea and juices are substitutes for ASBs, it is counter intuitive to think milk and ASBs are used in combination with each other as complementarity usually implies. This negative cross-price elasticity may be perhaps a reflection of the fact that milk is effectively a necessity with more than 80% of the households in the sample consuming it. Hence it is not unusual that price changes of ASB have a negative impact on the consumption of milk and vice versa.

The ASB consumption prevalence in India is 10 times higher among the rich households compared to the poor. While at least 10% of high-income households consumed ASBs, only about 1% of low-income households did so. The own-price elasticity reflected this strong income gradient with an elasticity coefficient of − 1.04 for the low-income households and − 0.83 for the high-income households. While the overall price elasticity of − 0.94 in our study is identical to the estimate from the only previous study [23] in India, the estimate by income group in our study varies. According to the previous study, the own-price elasticity ranged from − 0.9 to − 0.96 with poor households having relatively more inelastic demand which is counter-intuitive. Our study, on the other hand, shows poor has a relatively more elastic demand for ASBs compared to the rich in India. Moreover, the variation in the own-price elasticity coefficient in our study is larger between income groups. The failure to correct for the measurement errors and quality shading in unit values while using them as proxies for prices may have resulted in biased price elasticity estimates in the previous study.

The analysis of the trends in affordability of ASBs show that there has been an annual average decline of about 6.8% in RIP of ASBs in the past 13 years suggesting a steady increase in affordability. It was found that both decrease in real price and increase in income contributed to rising affordability in most years. A tax simulation using the estimated own-price elasticities reveal that a 29% compensation cess instead of the current 12% under the GST would result in a 10% reduction in ASB consumption, assuming a 100% tax pass-through.

Increased taxation has been a recommended and cost-effective policy option to regulate the use of ASBs and improve public health. The WHO report on *Fiscal Policies for Diet and Prevention of NCDs* [44] concludes there is reasonable and increasing evidence that appropriately designed taxes on ASBs aimed at raising the retail price by 20% or more would result in proportional reductions in consumption. In its report of the Commission on Ending Childhood Obesity too, the WHO has called for implementing an effective tax on ASBs [45]. Moreover, the Lancet Taskforce on NCDs and economics highlighted “the role of fiscal policies in encouraging healthy diets and lifestyles to reduce the largest contributors to preventable NCDs” [46]. The 2019 Task Force on Fiscal Policy for Health finds that “if all countries increased their excise taxes to raise prices on tobacco, alcohol, and sugary beverages by 50%, over 50 million premature deaths could be averted worldwide over the next 50 years while raising over $20 trillion of additional revenues in present discounted value” [47]. As many as 27 countries, including Portugal, Brunei, Saudi Arabia, Thailand, Mexico, United Kingdom, Ireland, South Africa, and the Philippines [1, 30, 34, 48–50] have either already implemented or are actively considering taxes on ASBs. It is also estimated that a 20% ASB tax would reduce overweight and obesity prevalence by 3.0% and type 2 diabetes incidence by 1.6% among various Indian subpopulations over the period 2014–2023 [23]. These experiences underscore the importance of using tax as an effective fiscal policy tool to regulate the consumption of ASBs.

This study suffers from some limitations. First, the estimate of price elasticity uses self-reported data on quantities and expenditures at the household level. Although it corrects for measurement errors and quality shading in unit values, the estimated elasticities are at the household level. Nevertheless, since ASBs are usually products consumed by most members of a household, it may not be inappropriate to consider a household as the basic unit of analysis. Second, the retail price data used for the affordability analysis are the ones collected for the consumer price index for industrial workers (CPI-IW) and its coverage of rural areas in India may be limited. To this extent, the average prices used for the affordability analysis may not be truly representative. However, since the objective is to examine the trends in average prices and not their absolute values, this limitation might not significantly affect our conclusions. Third, some of the aerated drinks included in our study may not necessarily have added sugar in it. The suggestions on taxation, however, are for the ASBs including these products too. A price increase in ASBs may result in increased demand for juices although the cross-price elasticities were not statistically significant and some of the juices may have added sugar present too. It would be better to have a product classification that distinguishes between drinks with and without added sugar and frame tax policies accordingly. However, the secondary data on consumption of soft drinks used in this study does not allow such disaggregation. Fourth, in the absence of reliable data on household income, total consumption expenditure is used as a proxy as is the convention in many studies. Hence, the accuracy of the grouping of income tertiles is subject to the quality of this proxy itself. Notwithstanding these caveats, this study provides a robust empirical evaluation of the price elasticity of ASBs and their affordability in India.

## Conclusion

Aerated or sugar-sweetened beverages are calorically dense and have little or no nutritional value [17]. People generally do not reduce their consumption of other calories after drinking ASBs, thus increasing the amount of excess energy consumed [18]. The evidence presented in this study shows that the prevalence of ASB consumption is nearly ten times higher among the rich compared to the poor. It implies that tax increases on ASBs will not be regressive as much of the tax burden on ASBs would go to relatively higher-income households. This study also shows that ASB consumers do respond to price increases. Hence taxation can be an effective tool to raise the price of ASBs, make it less affordable and thereby reduce its consumption. The taxation policy on ASBs in India, however, has been largely ineffective at increasing the real prices or reducing the affordability of ASBs as this study shows. An increased tax on ASBs is justified as it is an unhealthy product whose consumption needs to be curbed from a public health perspective. ASBs should be classified along with other unhealthy products like tobacco and alcohol as *demerit products* for taxation. The GST Council must regularly increase tax on ASBs enough to decrease its affordability with the primary purpose of regulating its consumption. It warrants a magnitude of tax increase such that it more than compensates for both general price inflation and income growth. This will essentially be a win-win situation as increased taxes while helping to decrease consumption and increase associated health gains, will bring in more tax revenue as the simulations in this study shows.

## Data Availability

All data used in this paper are secondary data that are freely available in the public domain. The NSSO household consumption data for sugar sweetened beverages which was used for the price elasticity estimates are available at http://microdata.gov.in/nada43/index.php/catalog/CEXP. Retail price data for the three aerated drink brands that was used to estimate affordability is available at http://labourbureaucpi.gov.in/.

## Abbreviations

ASB: Aerated or sugar-sweetened beverages
NCDs: Non-communicable Diseases
GST: Goods and Services Tax
NSO: National Statistical Office
RIP: Relative income price
GDP: gross domestic product
RBI: Reserve Bank of India
VAT: value-added tax
CBIC: Central Board of Indirect Taxes and Customs
